# The PPAR-γ Agonist Pioglitazone Modulates Proliferation and Migration in HUVEC, HAOSMC and Human Arteriovenous Fistula-Derived Cells

**DOI:** 10.3390/ijms24054424

**Published:** 2023-02-23

**Authors:** Carmen Ciavarella, Ilenia Motta, Francesco Vasuri, Teresa Palumbo, Anthony Paul Lisi, Alice Costa, Annalisa Astolfi, Sabrina Valente, Piera Versura, Eugenio F. Fornasiero, Raffaella Mauro, Mauro Gargiulo, Gianandrea Pasquinelli

**Affiliations:** 1 DIMEC—Department of Medical and Surgical Sciences, University of Bologna, 40138 Bologna, Italy; 2 Center for Applied Biomedical Research (CRBA), University of Bologna, 40138 Bologna, Italy; 3 Pathology Unit, IRCCS Azienda Ospedaliero-Universitaria di Bologna, 40138 Bologna, Italy; 4 Alma Mater Institute on Healthy Planet, University of Bologna, 40138 Bologna, Italy; 5 Department of Pharmacology & Physiology, Drexel University College of Medicine, 245 N. 15th Street, Philadelphia, PA 19102, USA; 6 Ophtalmology Unit, IRCSS Azienda Ospedaliero-Universitaria di Bologna, 40138 Bologna, Italy; 7 Department of Neuro-Sensory Physiology, University Medical Center Göttingen, 37073 Göttingen, Germany; 8 Department of Life Sciences, University of Trieste, 34127 Trieste, Italy; 9 Vascular Surgery Unit, IRCCS Azienda Ospedaliero-Universitaria di Bologna, 40138 Bologna, Italy

**Keywords:** intimal hyperplasia (IH), arteriovenous fistula (AVF), proliferation, migration, PPAR-γ, pioglitazone, HUVEC, HAOSMC, primary arteriovenous fistula cell model (AVFCs)

## Abstract

The failure of arteriovenous fistulas (AVFs) following intimal hyperplasia (IH) increases morbidity and mortality rates in patients undergoing hemodialysis for chronic kidney disease. The peroxisome-proliferator associated receptor (PPAR-γ) may be a therapeutic target in IH regulation. In the present study, we investigated PPAR-γ expression and tested the effect of pioglitazone, a PPAR-γ agonist, in different cell types involved in IH. As cell models, we used Human Endothelial Umbilical Vein Cells (HUVEC), Human Aortic Smooth Muscle Cells (HAOSMC), and AVF cells (AVFCs) isolated from (i) normal veins collected at the first AVF establishment (T0), and (ii) failed AVF with IH (T1). PPAR-γ was downregulated in AVF T1 tissues and cells, in comparison to T0 group. HUVEC, HAOSMC, and AVFC (T0 and T1) proliferation and migration were analyzed after pioglitazone administration, alone or in combination with the PPAR-γ inhibitor, GW9662. Pioglitazone negatively regulated HUVEC and HAOSMC proliferation and migration. The effect was antagonized by GW9662. These data were confirmed in AVFCs T1, where pioglitazone induced PPAR-γ expression and downregulated the invasive genes SLUG, MMP-9, and VIMENTIN. In summary, PPAR-γ modulation may represent a promising strategy to reduce the AVF failure risk by modulating cell proliferation and migration.

## 1. Introduction

The arteriovenous fistula (AVF) is the choice procedure for vascular access in patients subjected to hemodialysis for chronic kidney disease (CKD), whose incidence is increasing worldwide and represents a risk factor for cardiovascular disease (CVD) [1]. Despite the higher patency rate and the lower risk of complications in comparison to the other vascular access options, AVF carries a high percentage of failure because of inadequate maturation or degeneration, resulting in increased morbidity and further surgical operations [2,3,4,5]. AVF degeneration is commonly caused by neointimal hyperplasia (IH), a complex process characterized by progressive intimal thickening and consequent stenosis with lumen occlusion [3,6]. Shear stress and inflammation have been proposed as typical triggering events in the IH development [7,8]. IH pathogenesis involves different steps, starting from endothelial cell (ECs) damage and dysfunction [9], inflammation and smooth muscle cell (SMCs) phenotype transdifferentiation, proliferation and migration, culminating in vascular remodeling and matrix deposition (including calcification) [6]. CKD accelerates these processes, as observed in mice where it promoted endothelial dysfunction and neointimal formation in association with a lower expression of the junction protein Vascular Endothelial (VE)-Cadherin [10]. It has recently been proposed that endothelial-to-mesenchymal transition (End-MT) plays a pathogenic role in cardiovascular diseases, such as atherosclerosis [11] and plaque calcification [12]. End-MT is a phenotype conversion involving ECs that progressively lose the typical endothelial markers (i.e., CD31 and VE-Cadherin), shapes, and features, transiting into mesenchymal cells characterized by specific markers, morphology, and function [13]. A study performed on human atherosclerotic plaques and porcine aortas demonstrated that End-MT, triggered by shear stress, contributes to IH development [14].

The peroxisome-proliferator-associated-receptor-γ (PPAR-γ) belongs to a family of nuclear receptors, involved in the regulation of a broad range of biological mechanisms. Primarily, PPAR-γ is well known as a master regulator of the adipogenic differentiation process, by stimulating pre-adipocyte differentiation in mature adipocytes [15,16]. PPAR-γ also regulates glucose metabolism [17] and a class of PPAR-γ-agonists, the thiadolidondines, which is commonly used for type 2-diabetes treatment [18]. Additionally, PPAR-γ plays a pivotal function in regulating other biological processes, including cell proliferation and migration. In particular, it has been shown that PPAR-γ exerts a protective role in atherosclerosis, acting on vascular SMCs, ECs, and inflammatory cells. For this reason, the use of PPAR-γ agonists, such as pioglitazone or rosiglitazone, have been proposed for the therapy of cardiovascular diseases. However, the role of PPAR-γ in the progression of IH contextually to AVF failure has not been fully elucidated.

Here, we investigated the expression of PPAR-γ and the effects exerted by pioglitazone in cell populations mostly involved in IH pathogenesis, i.e., ECs and SMCs. Further, we established and characterized a model of primary cells isolated from veins subjected to the AVF procedure in order to investigate the nature of cells within the IH lesion and to reproduce the pathological context in vitro.

## 2. Results

### 2.1. Patient Characteristics

The present study included 12 end-stage kidney disease patients subjected to AVF surgery, distinguished in two groups (T0: normal veins collected at the first AVF establishment, including 3 males and 3 females, mean age 71 ± 13.7; T1: failed AVF with IH, including 3 males and 3 females, mean age 65 ± 12). The groups were homogenous for sex and age. Obesity, dyslipidemia, and diabetes mellitus were present in 14%, 42.8%, and 43% of all cases, respectively. Hypertension occurred in all cases, and tobacco smoke risk factor was recorded for the 43% of the patient group. 

### 2.2. Altered Distribution and Expression of PPAR-γ in Failed AVF Veins and Cells

**Figure 1:**
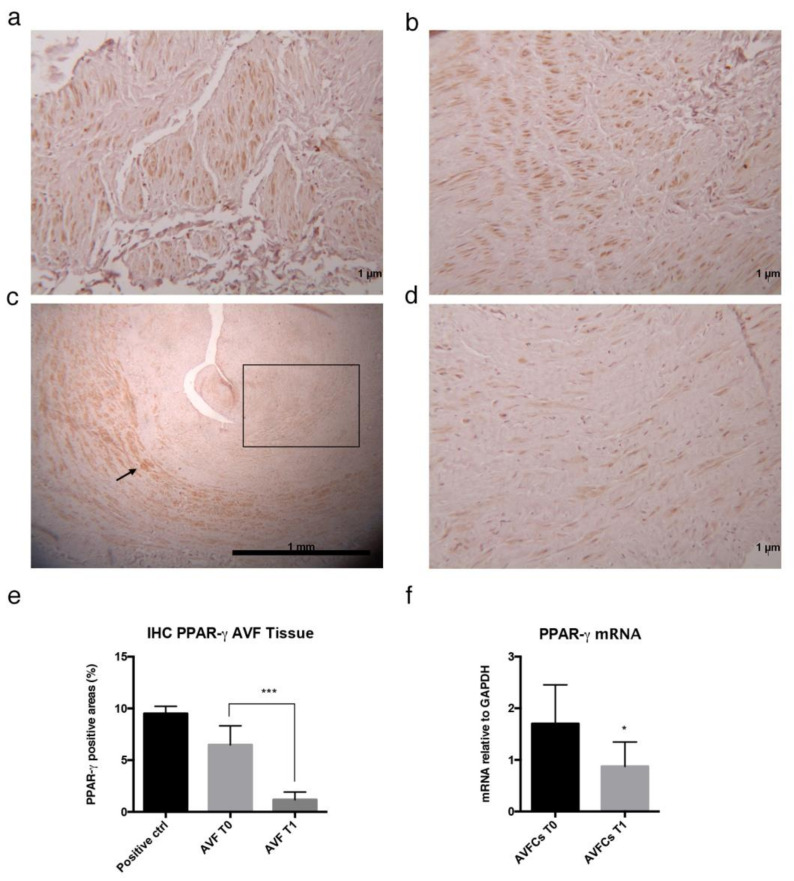
PPAR-γ expression in AVF tissues and cells. Immunohistochemical analysis of PPAR-γ in (**a**,**b**) normal AVF T0 and (**c**,**d**) failed AVF T1 veins. PPAR-γ protein was identified in AVF T0 tissues, localizing mainly in SMCs within the tunica media. In AVF T1 SMCs positive to PPAR-γ were detected in the tunica media of normal tissue surrounding the IH lesion but not directly involved in it (black arrows), whereas the IH characterized by the typical signs of intimal occlusion (black rectangle) did not express PPAR-γ. Mouse brown fat tissue was used as a positive control. (**e**) Quantification of PPAR-γ expressed as percentage of positive areas calculated on random fields captured from 10× images, by application of ImageJ (version 1.53) color deconvolution tool. (**f**) Expression of PPAR-γ mRNA in AVF cells (AVFCs T0 and AVFCs T1) analyzed by Real Time PCR. Results are shown as fold changes in AVFCs T1 relative to AVFCs T0. Each experiment was executed in triplicate (*n* = 3). Statistical analysis was performed by unpaired *t* test; * *p* = 0.0327; *** *p* = 0.0002. Abbreviations: AVF, arteriovenous fistula; AVFCs, arteriovenous fistula cells; *n*, number of values; PPAR-γ, peroxisome-proliferator-associated-receptor-γ.

In order to explore whether PPAR-γ could be a good candidate target for IH modulation and AVF failure prevention, we analyzed protein expression and localization in AVF tissues. Based on the histological findings, we observed an altered expression and distribution of PPAR-γ in failed AVF (T1). PPAR-γ was broadly expressed in native veins T0, mostly in SMCs within the tunica media (Figure 1a,b), whereas a differential expression pattern was highlighted in failed AVF. As evidenced in Figure 1c, PPAR-γ was not completely absent in the AVF veins and positive areas were detectable within the tissue surrounding the IH lesion. Conversely, all the areas interested by the IH lesion were negative to protein expression (Figure 1d). The percentage of PPAR-γ positive areas was assessed by applying the ImageJ deconvolution color tool on random fields in 10× images, confirming a lower percentage of receptor expression in AVF T1 veins (Figure 1e, percentage of PPAR-γ positive areas: 6.48 ± 0.97 in AVF T0 and 1.18 ± 0.29 in AVF T1; *p* value 0.0002, unpaired *t*-test). This PPAR-γ distribution pattern suggests the possible involvement of this transcription factor during the IH disease development and progression through the unbalanced production and function of PPAR-γ in proliferating cells that characterize the IH lesion. This result also found confirmation in mRNA analysis performed in the AVF cell model, where AVFCs T1 exhibited lower transcript levels of PPAR-γ (a 50% reduction) when compared to AVFCs T0 (Figure 1f). 

### 2.3. The PPAR-γ Agonist Pioglitazone Affects Proliferation and Migration Property in Endothelial and Smooth Muscle Cells

In order to test whether endothelial and smooth muscle cells, the key players during IH initiation and progression, were sensitive to PPAR-γ modulation through pharmacological approaches, we analyzed the effects of pioglitazone in HUVEC and HAOSMC. 

**Figure 2:**
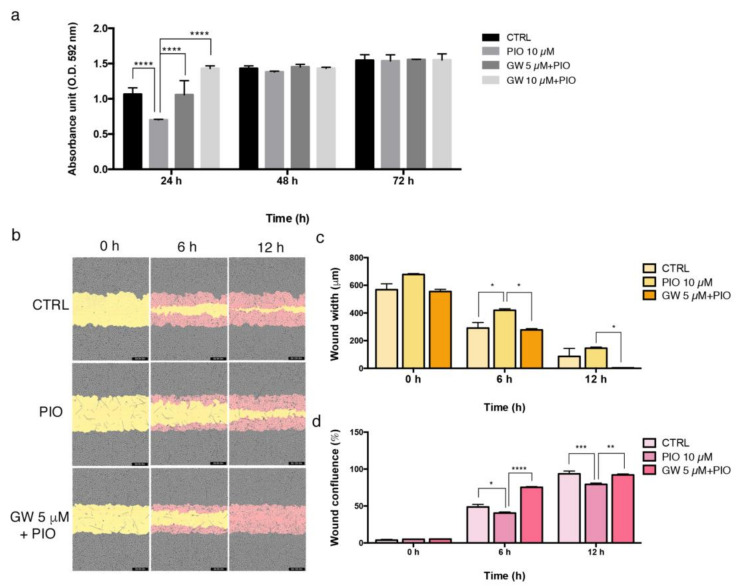
Pioglitazone modulates HUVEC proliferation and migration. (**a**) Analysis of HUVEC proliferation performed through crystal violet stain and expressed as absorbance values read with a Spark multimodal microplate reader at 595 nm. Results are reported as normalized to control with vehicle (DMSO) at 24 h. (**b**) Analysis of HUVEC migration was performed through wound scratch assay by using an Incucyte S3 instrument. Representative images of wound healing taken at 0 h (initial wound process), 6 h, and 12 h, acquired with a 10× objective lens. Yellow: initial scratch wound area; pink: HUVEC migrated into the scratch area. 10× magnification. Measurement of migration parameters were (**c**) wound width, expressed in micrometers (μm) and (**d**) wound confluence, expressed as a percentage (%). Each experiment was executed in triplicate (*n* = 3). Statistical analysis was performed by two-way ordinary Anova test, followed by Tukey’s multiple comparisons tests; * *p* < 0.05; ** *p* < 0.01; *** *p* < 0.001; **** *p* < 0.0001. Abbreviations: DMSO, dimethyl sulfoxide; GW, GW9662; HUVEC, Human Umbilical Vein Endothelial Cells; *n*, number of values; OD, optical density; PIO, pioglitazone.

According to the proliferation assay performed through crystal violet staining, we observed differences in dose/time response of HUVEC and HAOSMC to pioglitazone. In detail, HUVEC growth underwent a 30% decrease when exposed to pioglitazone at 10 μM for 24 h. This effect was prevented when pioglitazone was co-administrated with the PPAR-γ inhibitor GW9662 at 5 μM and 10 μM (Figure 2a). HUVEC treated with pioglitazone displayed increased expression of PPAR-γ protein and a down-regulation of nuclear factor kappa-light-chain-enhancer of activated B cells (NF-kB) and tumor necrosis factor α (TNF-α), both target genes of PPAR-γ and early regulators of the inflammatory process (Figure S1a,b). Pioglitazone also partially inhibited HUVEC migration potential, as shown by scratch wound assay performed through an Incucyte S3 assay (Figure 2b; see methods for experimental details). Data were analyzed at 6 h and 12 h post-wounding, showing that HUVEC were able to almost completely close the wound as evidenced by wound width reduction and wound confluence increase (Figure 2c,d). Pioglitazone significantly slowed down the wound closure process, whereas the GW9662 addition at 5 μM concentration contributed to reactivation of the migration features of HUVEC, coherently with wound width and wound confluence values (Figure 2c,d).

**Figure 3:**
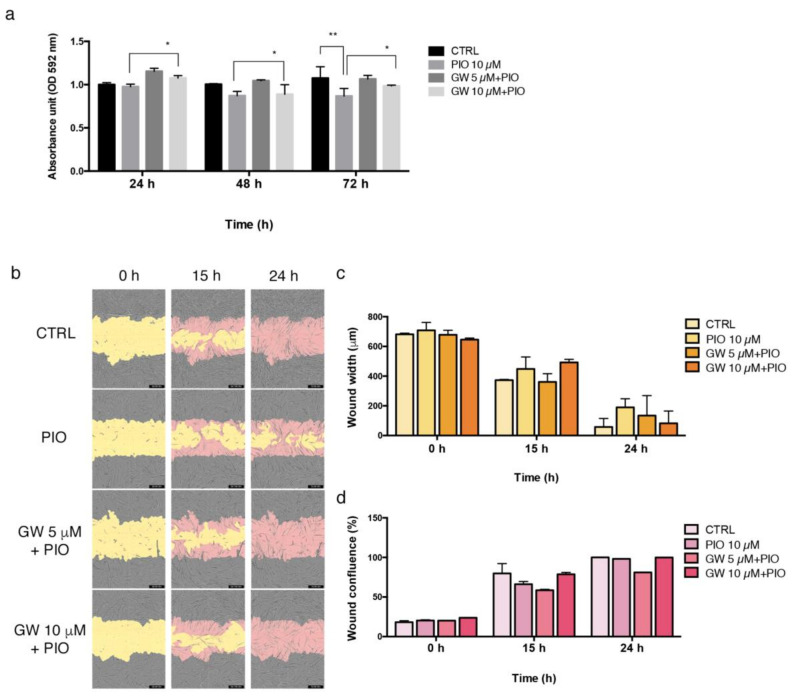
Pioglitazone modulates HAOSMC proliferation and migration. (**a**) Analysis of HAOSMC proliferation performed through crystal violet stain and expressed as absorbance values red by Spark multimodal microplate reader at 595 nm. Results are reported as normalized to control with vehicle DMSO at 24 h. (**b**) Analysis of HUVEC migration performed through wound scratch assay by using Incucyte S3 instrument. Representative images of wound healings taken at 10× objective lens 0 h (initial wound process), 15 h, and 24 h. Yellow: initial scratch wound area; pink: HAOSMC migrated into the scratch area. 10× magnification. Measurement of migration parameters (**c**) wound width, expressed in micrometers (μm) and (**d**) wound confluence, expressed as percentage (%). Each experiment was executed in triplicate (*n* = 3). Statistical analysis was performed by two-way ordinary Anova test, followed by Tukey’s multiple comparisons tests; * *p* < 0.05; ** *p* < 0.01. Abbreviations: OD, optical density; GW, GW9662; HAOSMC, human aortic smooth muscle cells; n, number of values; PIO, pioglitazone.

Similarly to HUVEC, pioglitazone affected HAOSMC proliferation by reducing cell growth, especially after 72 h administration (20% growth decrease; Figure 3a). PPAR-γ inhibition with GW9662 mitigated the effect of pioglitazone at each time point, more significantly at 10 μM. The analysis of cell migration evidenced a reducing effect mediated by pioglitazone after 15 h and 24 h from initial scratch (Figure 3b). GW9662 antagonized the effect of pioglitazone and the wound healing capacity of HAOSMC was restored (mainly after 15 h with GW9662 at 5 μM and after 24 h with GW9662 at 10 μM) (Figure 3b). These results were supported by measures of wound width and wound confluence reported in Figure 3c,d.

### 2.4. Characterization of a Primary Cell Model Isolated from Failed AVFs 

**Figure 4:**
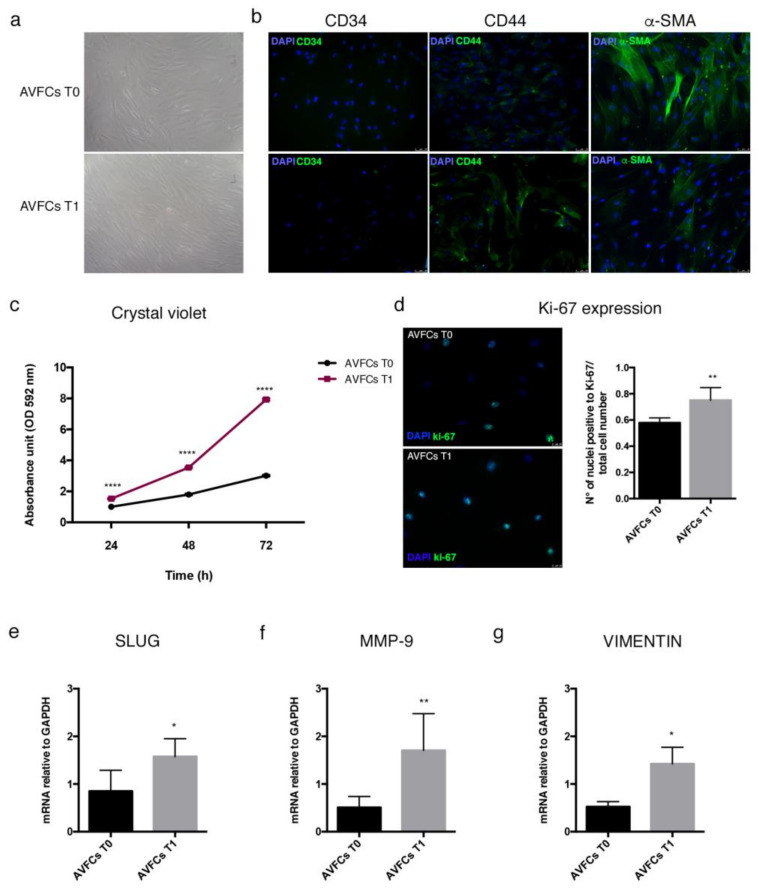
AVFCs T1 isolated from failed AVF exhibit a more proliferative and migratory phenotype than AVFCs T0. (**a**) AVFCs T0 and AVFCs T1 were adherent cells with fibroblast-like morphology (20× magnification), and (**b**) exhibited an immunophenotype negative to CD34 and positive to CD44 and α-SMA. Scale bar: 50 μm, 20× magnification. (**c**) AVFCs T1 displayed an increased proliferative curve, as shown by crystal violet stain of cells cultured until three days. Data are reported as mean ± standard deviation. (**d**) Representative immunofluorescence of ki-67 expression in AVFCs T0 and AVFCs T1, and relative quantification measured as ratio of ki-67 positive cell number on total cells. Green: ki-67, blue: DAPI. Scale bar: 25 μm, 40× magnification. Real-time PCR analysis of remodeling and migration genes (**e**) SLUG, (**f**) MMP-9, and (**g**) VIMENTIN. Results are expressed as fold changes, and a statistical analysis was performed between AVFCs T0 and AVFCs T1. Each experiment was executed in triplicate (*n* = 3). Statistical analysis was performed by two-way Anova in (**c**) and unpaired Student *t*-test in (**d**–**g**). * *p* < 0.05; ** *p* < 0.01; **** *p* < 0.0001. Abbreviations: AVFCs, arteriovenous fistula cells; MMP-9, matrix metalloproteinase 9; n, number of values; OD, optical density; α-SMA, smooth muscle actin alpha; SLUG, snail family transcriptional repressor 2 (SNAI2).

AVFCs T0 and AVFCs T1 displayed comparable morphology and immunophenotype, including the fibroblast-like morphology, the expression of CD44, α-smooth muscle actin (α-SMA), and the absence of endothelial antigen CD34, coherently with a mesenchymal stem cell (MSC)-like profile (Figure 4a,b). We did not observe significant variations of mesenchymal marker expression between T0 and T1. Conversely, a lower expression of α-SMA was observed in AVFCs T1 compared to AVFCs T0 (Figure 4b), suggesting the phenotype transition is occurring contextually to the IH development. In addition, the analysis of cell proliferation also highlighted divergence between AVFCs T0 and T1 cells. Indeed, AVFCs T1 exhibited higher growth rate than AVFCs T0, as supported by crystal violet stain (Figure 4c) and count of Ki-67 positive cells (Figure 4d). 

To further characterize the AVFC model with respect to the pathogenic processes typically associated with the IH development, we proceeded with the analysis of genes involved in vascular remodeling, cell migration, and invasion. AVFCs T1 displayed increased expression of matrix metalloproteinase 9 (MMP-9), snail transcription factor 1 (SLUG), and VIMENTIN than AVFCs T0 (Figure 4e–g). 

Altogether, these data were coherent with the unbalanced proliferative and migratory features of cells involved in IH pathogenesis. 

### 2.5. Pioglitazone Mitigated IH Pathogenic Mechanisms in AVF T0 and T1 Cell Model

**Figure 5:**
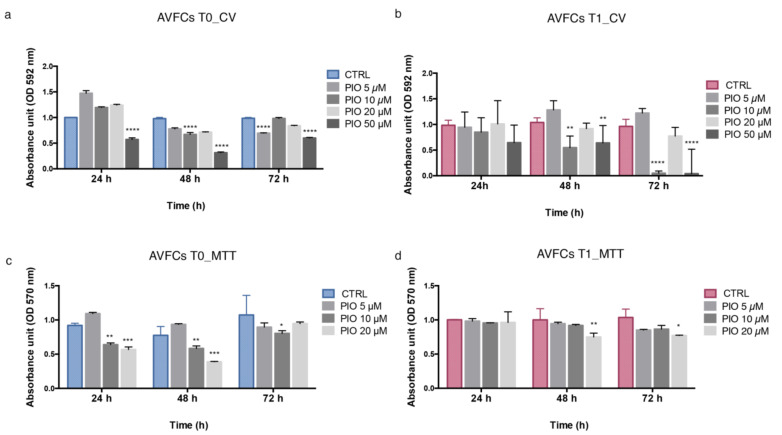
Pioglitazone determines proliferation decrease in AVFCs T0 and AVFCs T1. Analysis of AVFCs T0 (**a**,**b**) T1 proliferation evaluated by crystal violet (CV) stain and quantified in terms of absorbance by the Spark multimode microplate reader (Tecan) at 592 nm optical density (OD). Analysis of AVFCs T0 (**c**) and T1 (**d**) metabolic activity detected by MTT assay and quantified in terms of absorbance by the Spark multimode microplate reader (Tecan) at 570 nm OD. Number of values = 6. Data were normalized to untreated controls at 24 h, and analyzed by two-way Anova test with Tukey’s multiple comparisons tests. * *p* < 0.05; ** *p* < 0.01; *** *p* < 0.001; **** *p* < 0.0001. Abbreviations: AVFCs, arteriovenous fistula cells; CTRL, control; CV, crystal violet; MTT, 3-(4,5-dimethylthiazol-2-yl)-2,5-diphenyl -2H-tetrazolium bromide; OD, optical density; PIO, pioglitazone.

Preliminary results showed a lower expression of PPAR-γ in AVFCs T1 than T0, suggesting a possible regulatory role during IH pathogenesis. In order to explore whether PPAR-γ stimulation could mitigate IH mechanisms, both AVFCs T0 and AVFCs T1 were exposed to pioglitazone at increasing concentrations (0–5–10–20–50 μM). Pioglitazone affected AVFCs proliferation in a dose- and time-dependent manner, especially in AVFCs T0, coherently with the unbalanced proliferative phenotype in AVFCs T1 cells (Figure 5a). However, we did not observe detrimental effects on cell viability in the presence of low drug concentrations (5–10 μM) for 24–48 h. In particular, we found a significant growth decrease in cells treated with 10 μM pioglitazone for 48 h (49.1% ± 0.225 in AVFCs T1, *p* < 0.01; 31% ± 0.036 in AVFCs T0, *p* < 0.0001; two-way ordinary Anova test). More drastic effects on cell viability were detected at higher pioglitazone doses and at prolonged exposures (20–50 μM), especially in AVFCs T1 (Figure 5b). The analysis of the cell metabolic activity performed by MTT assay supported the proliferation results. In AVFCs T0, the resulting amount of cell viability and metabolic activity was reduced by 25% and 18 % after 24 h and 48 h, respectively (Figure 5c). In AVFCs T1, the metabolic activity was not drastically affected at 10 μM for 24 h and 48 h (Figure 5d). Based on these data, the pioglitazone administration at 10 μM for 48 h resulted in more effective experimental conditions to modulate cell proliferation without excessive cytotoxic effects. 

In addition, pioglitazone affected the cell migration process in AVFCs T0 and AVFCs T1, as detected by the wound healing assay performed by a manual scratch test (Figure S2a). 

**Figure 6:**
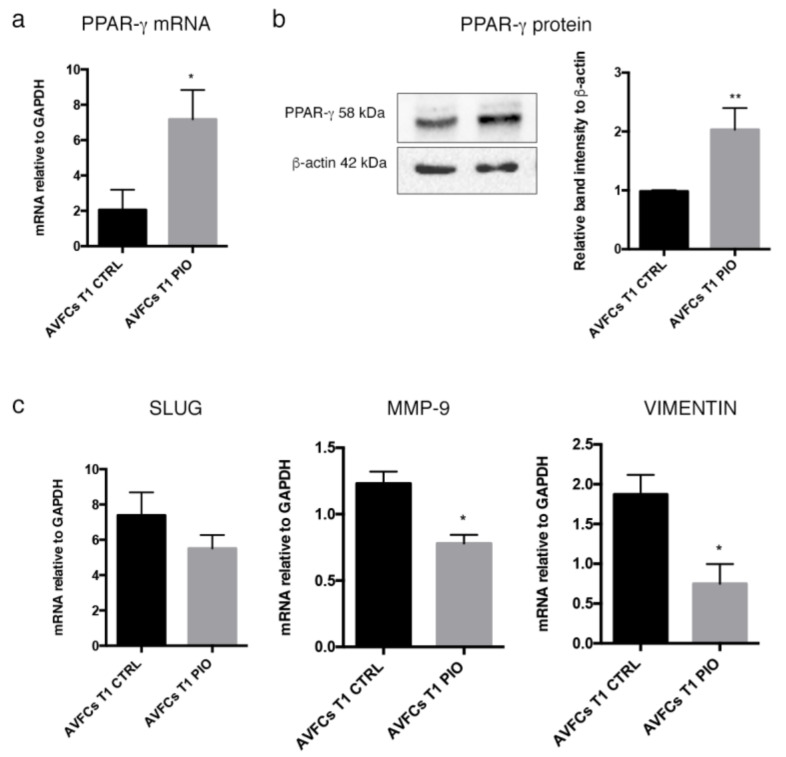
Pioglitazone induces PPAR-γ expression and down-regulates migration/invasion genes in AVFCs T1. (**a**) *PPAR-γ* mRNA expression by real-time PCR and (**b**) representative Western Blot image (**left**) and densitometric analysis (**right**) of PPAR-γ protein expression in AVFCs T1 following treatment with pioglitazone at 10 μM for 48 h, corresponding at the experimental setting associated with growth cell decrease. (**c**) mRNA analysis of *SLUG*, *MMP-9*, and *VIMENTIN* genes in AVFCs T1 following PPAR-γ induction by pioglitazone. Real-time results are expressed as fold changes relative to untreated controls. All data are reported as mean ± SEM of at least three independent experiments (*n* = 3). Statistical analysis was performed by paired Student’s *t*-test relative to untreated controls. * *p* < 0.05; ** *p* < 0.01. Abbreviations: AVFCs, arteriovenous fistula cells; CTRL, control; GAPDH, glyceraldehyde 3-phospate dehydrogenase; n, number of values; PIO, pioglitazone; PPAR-γ, peroxisome-proliferator-associated-receptor-γ.

Next, we explored whether pioglitazone at the selected concentration was able to stimulate PPAR-γ expression in AVFCs T1. It was found that a significant up-regulation of *PPAR-γ* mRNA and protein in AVFCs T1 treated with pioglitazone at 10 μM for 48 h (Figure 6a,b) and in AVFCs T0 (Figure S2b). These data showed that AVFCs T1 resulted in being more sensitive to PPAR-γ modulation by pioglitazone, confirming PPAR-γ as a potential target for regulating the increased proliferative process occurring in IH. Further, coherently with the ameliorating effect on cell migration, pioglitazone also mediated the down-regulation of the main genes involved in cell migration/invasiveness SLUG, MMP-9, and VIMENTIN in AVFCs T1 (Figure 6c). 

### 2.6. PPAR-γ Inhibition Reversed the Regulatory Effects of Pioglitazone in AVFCs T1 Proliferation and Migration

**Figure 7:**
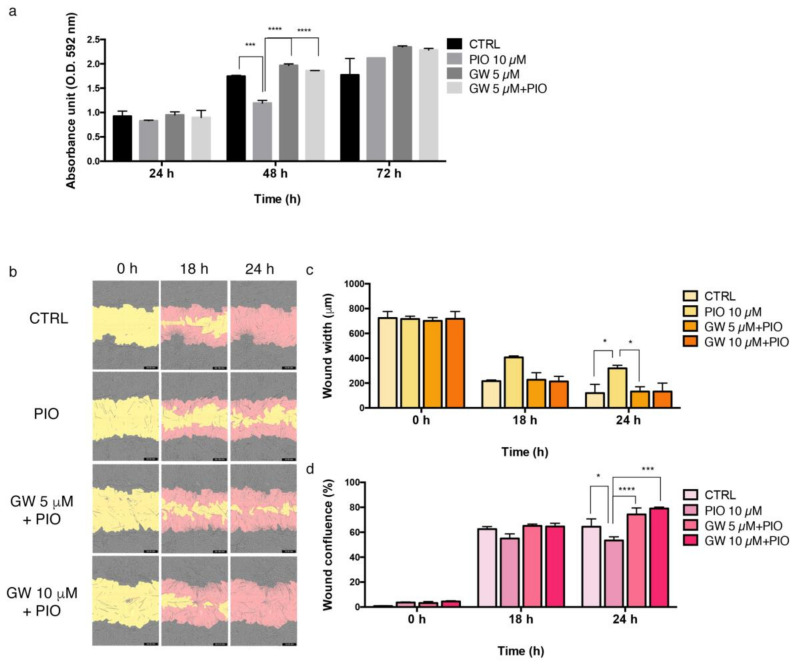
GW9662 reverses pioglitazone effects in AVFCs T1. (**a**) Analysis of cell proliferation in AVFCs T1 treated with pioglitazone (10 μM) alone or in combination with the PPAR-γ inhibitor GW9662 (5–10 μM). Absorbance values were analyzed by Spark multimode microplate reader (Tecan) at 592 nm OD and normalized to untreated control at 24 h. (**b**) Analysis of AVFCs T1 migration performed through wound scratch assay by using Incucyte S3 instrument. Representative images of wound healing were taken at 10× objective lens 0 h (initial wound process), 18 h and 24 h; yellow: initial scratch wound area, pink: AVFCs T1 migrated into the scratch area. Measurement of migration parameters (**c**) wound width, expressed in micrometers (μm) and (**d**) wound confluence, expressed as a percentage (%). Each experiment was executed in triplicate (*n* = 3). Statistical analysis was performed by two-way ordinary Anova test, followed by Tukey’s multiple comparisons tests; * *p* < 0.05; *** *p* < 0.001; **** *p* < 0.0001. Abbreviations: AVFCs, arteriovenous fistula cells; CTRL, control; GW, GW9662; n, number of values; OD, optical density; PIO, pioglitazone.

In order to confirm whether pioglitazone regulates IH pathogenesis via PPAR-γ stimulation, AVFCs T1 were exposed to pioglitazone/GW9662 combination. As shown in Figure 7, GW9662 reversed the effect of pioglitazone by reactivating the proliferative (Figure 7a) and migratory (Figure 7b) processes in AVFCs T1. An increasing trend of AVFCs T1 migration was observed with both GW9662 concentrations, particularly evident at 5 μM as highlighted by wound width (Figure 7c) and wound confluence (Figure 7d) measurements. These data supported the regulatory role of PPAR-γ on cell proliferation and migration mechanisms, which are crucial during IH pathogenesis. 

## 3. Discussion

IH development is the leading cause of inadequate maturation and consequent failure of AVF, resulting in increased cardiovascular complications and repeated interventions. Vascular damage, shear stress, and surgical trauma contribute to IH initiation, whose cellular and molecular mechanisms involve several cell players and have not been fully elucidated yet. IH is a neointimal lesion typically characterized by unbalanced proliferation and migration processes of vascular cells, including ECs and SMCs. The regulation of cell proliferation and migration represents a possible strategy to prevent IH and AVF failure.

With the intent of identifying a possible regulatory strategy of the IH pathogenesis, the present study investigated the involvement of the nuclear hormone receptor PPAR-γ in AVF through its modulation by the pharmacological agonist pioglitazone. As reported in the literature, PPAR-γ performs anti-proliferative functions in addition to the well-known metabolic regulatory roles [19], indeed PPAR-γ over-expression was shown to inhibit vascular SMC proliferation and IH in mice through the inhibition of the Toll-like Receptor 4 (TLR4)-mediated inflammation [20]. Further, the PPAR-γ agonist rosiglitazone was able to modulate rat aortic VSMCs and to reduce IH after angioplasty in a rat carotid artery model [21]. The PPAR-γ agonist pioglitazone is an insulin-sensitizing drug with anti-fibrotic and anti-inflammatory effects, able to exert beneficial effects in many cardiovascular pathological events by contributing to the reduction of atherosclerotic plaque, neointimal formation, plaque inflammation, and to the repair of endothelial function, as reviewed in Nesti et al. [22]. Pioglitazone also preserves beta-cell function and improves the metabolic syndrome through several functions, i.e., by enhancing insulin sensitivity, lowering blood pressure, reducing triglycerides and increasing high-density lipoprotein (HDL) (reviewed in DeFronzo et al. [23]). Like rosiglitazone, pioglitazone was effective at reducing IH, by promoting a significant regression of IH in balloon-injured rat carotid artery [24] and by mitigating the IH formation in mouse injured femoral artery [25]. 

The protein and mRNA analysis performed in veins collected contextually to first surgical procedure for AVF (T0) and repeated intervention due to AVF failure (T1), revealed an altered expression of PPAR-γ in failed AVF. In particular, the histological findings highlighted a differential pattern of localization of PPAR-γ protein in AVF T1, as it was normally expressed in the tissue surrounding the lesion and negative within the neointimal formation. Based on this preliminary and clinical sketch of PPAR-γ distribution in AVF tissues, we aimed at exploring whether its pharmacological modulation in vitro was able to counteract IH pathogenic processes. Firstly, we observed that HUVEC and HAOSMC, representative of endothelial and smooth muscle cell models, were highly responsive to pioglitazone administration undergoing growth decrease and wound healing property impairments. Interestingly, the co-administration of inhibitor/agonist of PPAR-γ (GW9662/pioglitazone) reversed this trend by reactivating both cell proliferation and migration. According to the literature [26,27], these data supported the role of PPAR-γ as potential modulator of vascular remodeling and pathological alterations. We therefore extended the analysis to a primary cell model isolated from AVF patients included in a group homogenous for age, sex, and risk factors. This model consisted of cells characterized by a mesenchymal-like phenotype, given by adherence to plastic, typical elongated fibroblast shape, expression of CD44, and lack of CD34, the endothelial cell marker. Further, we detected differential expression of α-SMA protein, which resulted lower in AVFCs T1 and possibly suggestive of altered differentiation lineage during IH progression. Cells were also characterized according to functional properties, displaying a more prominent growth rate and ki-67 expression and marker of proliferation in AVFCs T1 in comparison to AVFCs T0. This result was consistent with a previous study of our group, which elucidated the increased positivity to ki-67 in AVF tissues belonging to the T1 group [28]. Additionally, AVFCs T1 exhibited high mRNA levels of SLUG, MMP-9 and VIMENTIN, genes typically involved in migration, invasion, vascular remodeling, and End-MT. End-MT is a crucial phenotype conversion occurring in ECs in both physiological and pathological states, and might be a source of key cell contributors to vascular pathology, and, considering the IH localization within the intimal layer, to AVF failure. In this regard, it was demonstrated that blocking Notch signaling, responsible for End-MT activation in ECs, prevented AVF failure [29]. 

PPAR-γ was lower in AVFCs T1 than AVFCs T0, reflecting the histological expression pattern. Pioglitazone was effective at modulating cell proliferation in both AVFCs T0 and T1, particularly in AVFCs T1 where the decrease of growth rate was associated with the up-regulation of PPAR-γ expression. Pioglitazone also reduced AVFCs T1 migration and relative genes levels (SLUG, MMP-9, VIMENTIN). MMP-9 also represents a crucial factor in AVF failure, as shown by a model of AVF mice where stenosis and inflammation were reduced following MMP-9 knockout [30]. 

The exposure of AVFCs T1 to pioglitazone/GW9662 counteracted both the proliferation and migration rates observed in presence of pioglitazone alone. These results support the pivotal role of PPAR-γ in the resolution of IH pathogenic mechanisms in vitro, paving the way to further investigations aimed at identifying novel PPAR-γ target genes to be proposed in the prevention of IH and AVF failure. 

Future studies will be necessary to unveil alternative approaches involving natural compounds [19] to stimulate PPAR-γ expression and activity, in order to overcome side effects due to drug therapy such as fluid retention and the risk for heart failure associated with pioglitazone administration [22]. To this end, a recent study showed the efficacy of Jujuboside B, a saponin extracted from Zizyphus jujuba var. spinosa, at modulating VSMCs proliferation and migration, via AMPK/PPAR-γ signaling [31]. Similarly, fisetin, a plant flavonoid polyphenol, was found to inhibit VSMC proliferation and migration, and to ameliorate IH following injury, by inducing the antioxidant enzyme paraoxonase (PON2) through PPAR-γ activation [32].

Ultimately, a noteworthy feature of the present study is the utilization of cell models isolated from patients subjected to AVF intervention, with the goal to reproduce the disease context in vitro and to characterize the cell populations that participate to IH pathogenesis. However, the high level of biological variability existing among different patients affects data reproducibility and points out the importance of properly evaluating the treatment schedule taking into account of patient specific characteristic for more personalized and effective approaches. 

## 4. Materials and Methods

### 4.1. Study Design and Sample Collection

For the present study, vascular tissues were collected from end-stage kidney disease patients subjected to AVF surgical procedure, with the approval of the local Ethical Committee (protocol number 142/2019/Sper/AOUBo). All samples were anonymous and treated according to the ethical guidelines of the 1975 Declaration of Helsinki and following revisions. After surgery, fresh vein tissues were both fixed with formalin for histological analysis and processed for cell isolation, as described below in this section. Tissues were distinguished as follows: (1) T0, normal veins taken when the first AVF was established, and (2) T1, failed AVF with IH. 

### 4.2. Immunohistochemistry 

PPAR-γ expression was detected in AVF T0 and AVF T1 tissues by immunohistochemistry using a non-biotin-amplified method (Novolink, Leica Biosystems, Wetzlar, Germany). To this aim, 2 μm thick sections of formalin-fixed and paraffin-embedded tissues (FFPE) were deparaffinized and rehydrated through a series of graded ethanol and rinsed in distilled water. Inactivation of endogenous peroxidase activity was performed with a 3% H_2_O_2_ in absolute methanol solution for 10 min (min) at room temperature (rt), antigen retrieval was performed using citrate buffer (pH 6) in microwave, and after cooling, slides were washed with Tris Buffered Saline (TBS). Sections were incubated with PPAR-γ primary antibody (1:100, clone C26H12, Cell Signaling Technology, Danvers, MA, USA) in a moist chamber at 4 °C over/night (o/n), followed by NovoLink Polymer for 30 min at rt and finally exposed to the substrate/chromogen 3,3′-diaminobenzidine (DAB) Novocastra DAB Chromogen and NovoLink DAB buffer. Nuclei were counterstained with Mayer’s hematoxylin. Samples were dehydrated, cover slipped, and observed under a light microscope using the Image Pro Plus program. The quantification of PPAR-γ was performed on digitalized images randomly acquired at 10× magnification by using the Image J (version 1.53) deconvolution color tool. Results were expressed as mean of PPAR-γ-positive areas.

### 4.3. Cell Cultures and Treatments

Cell models used in this study were the following: human endothelial umbilical vein cells (HUVEC, Lonza, Basel, Switzerland), human aortic smooth muscle cells (HAOSMC, Promocell, Heidelberg, Germany), and primary cells isolated from veins. HUVEC were cultured in Dulbecco’s Modified Eagle’s Medium (DMEM) enriched with 10% fetal bovine serum (FBS) and 1% antibiotics (Euroclone, Milano, Italy). HAOSMC were cultured with HAOSMC growth medium (Promocell, Heidelberg, Germany). Primary cells were isolated from vascular tissue (T0 and T1) collected during AVF surgical procedure. The cell isolation procedure was performed through organ culture. To this scope, at time of tissue collection, AVF tissues were rinsed with Phosphate Buffer Saline (PBS, Merck Group, Darmstadt, Germany) and then placed in 12-well plates with 2 mL of DMEM enriched with 20% FBS and 1% antibiotics. Growth medium was freshly replaced twice a week and culture growth was observed daily under the inverted microscope. Tissue cultures were kept in incubator at 37 °C, 5% CO_2_ for two weeks, when cells started to grown from explant. After tissues were discarded, cells were cultured for further 10 days, when 70% confluence was reached. AVFCs were used at passages 3–10, for analysis of immunophenotype, proliferation, migration, and gene and protein expression. 

Pioglitazone hydrochloride (E6910; Merck Group, Darmstadt, Germany) and GW9662 (M6191; Merck Group, Darmstadt, Germany) were used as pharmacological agonist and irreversible inhibitor of PPAR-γ, respectively. For PPAR-γ inhibition, a preliminary exposure to GW9662 was performed for 30 min, followed by combination with pioglitazone. Experimental scheme (concentration/exposure time) was optimized according to viability assay for each cell model. Treatments were administrated with complete growth medium, and controls were grown with dymethyl sulfoxide (DMSO; Merck Group, Darmstadt, Germany), which was used as vehicle solvent for compound preparations.

### 4.4. Immunofluorescence

The analysis of AVF immunophenotype was performed through immunofluorescence. Cells were fixed with 2% paraformaldehyde (membrane antigens) for 4 min at rt. Then, cells were permeabilized with Triton X-100 (Merck Group, Darmstadt, Germany) at 1% in PBS for additional 10 min at rt for cytoplasmic and nuclear antigen detection. Incubation with bovine serum albumin (BSA, Merck Group, Darmstadt, Germany) 1% in PBS was assessed for 30 min at rt for the blocking of non-specific binding sites. Cells were incubated with primary antibodies (CD34 1:80, Dako, Santa Clara, CA, USA; CD44 1:100, BD Biosciences Pharmingen, Franknlin Lakes, NJ, USA; α-SMA 1:100, Sigma Aldrich, St Louis, MO, USA; Ki-67 1:100, Novocastra, Leica Biosystems, Wetzlar, Germany) for 1 h at 37 °C. Samples were then washed with PBS and incubated with anti-mouse Alexa Fluor 488 and anti-rabbit Alexa Fluor 546 (ThermoFisher Scientific, Carlsbad, CA, USA) secondary antibodies in 1% BSA/PBS for 1 h at 37 °C in the dark. After washing with PBS, nuclei were counterstained with DAPI (4′, 6-diamidino-2-phenylindole; Thermo Fisher Scientific, Carlsbad, CA, USA). Images were acquired by a Leica DMI4000 B inverted fluorescence microscope (Leica Microsystems, Milan, Italy). Quantification of Ki-67 positive cells was performed on digitalized images randomly acquired at 40× magnification, and a minimum of 5 fields was examined for each sample. Results were expressed as percentages of nuclei positive to the target protein and expressed as percentage of positive cells/total cells. 

### 4.5. Cell Growth and Proliferation Assay 

The analysis of cell proliferation was performed through crystal violet stain and MTT assay.

Crystal violet stain was assessed in HUVEC, HAOSMC, AVFCs T0, and AVFCs T1 for testing the effect of pioglitazone, alone and in combination with GW9662, on cell proliferation. Cells were seeded in a 96-well plate in triplicate in complete growth medium at different density seeding for each cell model (HUVEC 5 × 10^3^/well; HAOSMC 10 × 10^4^/well; AVFCs T0, T1: 10 × 10^4^/well). After 24 h, cells were fixed with formalin for 10 min at rt, washed with PBS, and stained with crystal violet for 20 min. Crystal violet excess was removed by four washes with distilled water, air dried, and solubilized with 10% acetic acid. The quantification of cell viability was performed by absorbance analysis at 592 nm optical density (OD) by Spark multimode microplate reader (Tecan, Zurich, Switzerland).

For metabolic activity analysis following pioglitazone administration, MTT assay (Vybrant MTT Cell Proliferation Assay Kit, Thermo Fisher Scientific, Carlsbad, CA, USA) was performed in AVFCs T0 and AVFCs T1, following the manufacturer’s instructions. Cells were seeded in a 96-well plate in triplicate at a density of 10^4^ cells/well in 100 μL complete growth medium. After 24 h, cells were treated with pioglitazone (0–5–10–20 μM) for 24–48–72 h. Then, cell medium was replaced with fresh growth medium and 10 μL of 12 mM MTT component A (3-(4,5-dimethylthiazol-2-yl)-2,5-diphenyltetrazolium bromide) were added to each well and left in incubator for 4 h. Then, 100 μL of MTT component B sodium dodecyl sulfate (SDS)-hydrochloride acid (HCl) were added for further 18 h at 37 °C. Absorbance was analyzed at 570 nm OD by a Spark multimode microplate reader (Tecan, Zurich, Switzerland).

### 4.6. In Vitro Migration Assay

The analysis of cell migration in HUVEC, HAOSMC, AVFCs T0, and AVFCs T1 under pioglitazone/GW9662 treatments was performed through a scratch wound assay. For manual scratch assay, 1 × 10^5^ AVFCs T0 and T1 were seeded in a 24-well plate. After 24 h, the cell monolayer was wounded with a sterile p200 pipette tip, washed with PBS, and treated with pioglitazone at 10 μM in DMEM 10% FBS. Cell migration was monitored under the light microscope until 48 h, when cells were fixed with formalin at rt, washed with PBS, stained with 0.1% Crystal Violet in 25% methanol for 25 min, and air-dried. Images were taken with a digital camera (Nikon). For automated scratch assay, cells (HUVEC, HAOSMC, AVFCs T1) were seeded in an Incucyte ImageLock 96-well plate (Essen Bioscience, Ann Harbor, MI, USA) and treated with pioglitazone alone or in combination with GW9662 according to the experimental design. The scratch was performed by using the WoundMaker (Essen BioScience, Ann Harbor, MI, USA) device. After a PBS wash, cell treatments were replenished and the plates were placed in an IncuCyte S3 instrument (Essen BioScience, Ann Harbor, MI, USA) equipped with a dedicated incubator. Then, each wound image per well was automatically recorded with a 10× objective lens every three h for 48 h using the IncuCyte S3/SX1 optical module phase contrast. Images were processed by using the IncuCyte 2022B software to analyze, over time, the wound width that is the distance between the migrating edges of the wound and measured in micrometers (μm), and the wound confluence, which represents the percentage (%) of the wound region occupied by cells. 

### 4.7. Gene Expression Analysis

Total RNA was extracted from AVF T0 and AVF T1 through PureZOL^TM^ RNA isolation reagent (BioRad Laboratories, Hercules, CA, USA), according to the manufacturer’s instructions. Reverse transcription was performed from 1 µg of total RNA in 20 µL reaction volume using iScriptTM cDNA synthesis kit (BioRad Laboratories, Hercules, CA, USA). Real-time PCR was carried out in a CFX Connect real-time PCR Detection System (BioRad Laboratories, Hercules, CA, USA) using the SYBR green mix (Sso AdvancedTM Universal Sybr Green Supermix; BioRad Laborato-ries, Hercules, CA, USA) and primers sequences were designed using the NCBI BLAST tool and purchased from Merck Group, Darmstadt, Germany (Table 1). Each assay was performed in triplicate and each target gene expression was normalized to glyceraldehyde 3-phospate dehydrogenase (GAPDH). Gene expression levels were determined by the comparative 2^−ΔΔCt^ method and expressed as fold changes relative to controls [33].

### 4.8. Western Blot

Total cellular proteins were extracted from AVFCs T1 treated with pioglitazone (10 μM) for 48 h using a lysis buffer (0.1 M KH2PO4, pH 7.5, 1% NP-40, 0.1 mM β-glycerolphosphate, supplemented with protease inhibitor cocktail; Sigma-Aldrich, St Louis, MO, USA) and quantified through the Bio-Rad Protein Assay (BioRad Laboratories, CA, USA) at the spectrophotometer. Thirty micrograms of proteins were separated on 10% polyacrylamide gel by SDS-PAGE (TGX Stain-Free™ FastCast™ Acrylamide Solutions; BioRad Laboratories, Hercules, CA, USA). Proteins were transferred to a nitrocellulose membrane (GE Healthcare Life Sciences, Chicago, IL, USA), blocked with 5% non-fat dry milk in TBS-Tween for 1 h at rt, and incubated with the primary antibodies PPAR-γ (1:1000; clone C26H12, Cell Signaling Technology, Danvers, MA, USA), β-actin (1:4000; clone AC-74, Sigma-Aldrich, St. Louis, MO, USA) at 4 °C o/n. Incubation with secondary antibody human anti-rabbit/mouse horseradish peroxidase-conjugated (GE Healthcare, Chicago, IL, USA) was performed for 1 h at rt. The protein signal was detected using Westar ηC chemiluminescent substrate (Cyanagen, Bologna, Italy). Membrane imaging and densitometric analysis were performed at the ChemiDoc XRS+ Imaging System (BioRad Laboratories, Hercules, CA, USA) using ImageLab v5.1.1 (BioRad Laboratories, Hercules, CA, USA).

### 4.9. Statistical Analysis

For each experiment, at least three biological and technical replicates were performed. Data were expressed as mean ± standard deviation (SD) or standard error of mean (SEM). Data analysis and graphs were developed with GraphPad Prism 6 statistical software. Statistical analysis was performed through unpaired and paired Student’s *t*-test for comparison between two groups, whereas ordinary one-way and two-way analyses of variance (ANOVAs) followed by Tukey’s test were applied for multiple comparisons. Results were considered statistically significant at the 95% confidence level (*p* < 0.05). 

## 5. Conclusions

In the present study, PPAR-γ induction by pioglitazone was performed in three different cell models, represented by HUVEC, HAOSMC, and patient AVFCs. The obtained data were coherent at supporting the PPAR-γ induction strategy as effective at inhibiting cell proliferation and migration, key mechanisms in IH development. These results can be preliminary to future studies addressed at investigating the PPAR-γ downstream signaling pathway and at searching for novel agonists to prevent IH and AVF failure.

## Figures and Tables

**Table 1:** List of primer sequences used for gene expression analysis. FWD, forward; GAPDH, glyceraldehyde 3-phospate dehydrogenase; MMP-9, matrix metalloproteinase 9; NF-kB, nuclear factor kappa-light-chain-enhancer of activated B cells; PPAR-γ, peroxisome-proliferator activated receptor γ; REV, reverse; SLUG, snail transcriptional repressor 2; TNF-α, tumor necrosis factor alpha.

Gene	Primer Sequence	
GAPDH	FWD 5′AATGGGCAGCCGTTAGGAAA 3′ REV 5′ AGGAGAAATCGGGCCAGCTA 3′	
MMP-9	FWD 5′ GAACCAATCTCACCGACAG 3′ REV 5′ GCCACCCGAGTGTAACCAT 3′	
NF-kB	FWD 5′ AGGCTATCAGTCAGCGCATC 3′ REV 5′ TCCCCACGCTGCTCTTCTAT 3′	
PPAR-γ	FWD 5′ GTGGTAGGTAAGGAAGGGGC 3′ REV 5′ GGCTGACTCTCGTTTGAGAA 3′	
SLUG	FWD 5′ TTCAACGCCTCCAAAAAGCC 3′ REV 5′ GATGGGGCTGTATGCTCCTG 3′	
TNF-α	FWD 5′ CAGGGACCTCTCTCTAATCA 3′ REV 5′ TTGAGGGTTTGCTACAACAT 3′	
VIMENTIN	FWD 5′ATCGATGTGGATGTTTCCAA 3′ REV 5′ TTGTACCATTCTTCTGCCTC 3′	

## Data Availability

Not applicable.

## Supplementary Materials

The following supporting information can be downloaded at: https://www.mdpi.com/article/10.3390/ijms24054424/s1.
