# Supplementary material for: The PPAR-γ Agonist Pioglitazone Modulates Proliferation and Migration in HUVEC, HAOSMC and Human Arteriovenous Fistula-Derived Cells
Source: Int J Mol Sci. 2023 Feb 23;24(5):4424. doi: 10.3390/ijms24054424 (PMC10003103; doi:10.3390/ijms24054424)
Supplement: Supplementary file 1 [file ijms-24-04424-s001.zip › ijms-2161770-supplementary.pdf]

Supplementary File

Western Blot for PPAR-γ detection in HUVEC

Total cellular proteins were extracted from HUVEC using Laemmli Sample Buffer (BioRad Laboratories, CA, USA) with β-mercaptoethanol. Samples were run in gel containing 10% polyacrylamide (Roth A124.2) and transferred to nitrocellulose blotting membrane (Amersham Protran Premium 10600003). Then, membrane was blocked with 5% non-fat dry milk in TBS-Tween for 1 h at room temperature and incubated with the following primary antibodies: rabbit anti-PPARγ (1:1000; C26H12, Cell Signaling Technology, Danvers, MA, USA) and mouse anti-GAPDH (1:5000; mouse anti-GAPDH (1:5000; 60004-1-Ig, Proteintech, Rosemont, IL, USA) for 1 h at room temperature and then washed with TBS-Tween for 30 minutes. Membrane was then incubated for 1h at room temperature with respective secondary antibodies: donkey anti-rabbit IgG antibody conjugated to IRDye 800 CW (1:5000; 926-32213, LI-COR, Lincoln, NE, USA) and donkey anti-mouse IgG antibody conjugated to IRDye 680RD (1:5000; 926-68072, LI-COR, Lincoln, NE, USA) and washed with TBS-Tween for 30 minutes. The protein signal was detected with a LI-COR Odyssey CLX-2088 imaging system.

Supplementary Figure S1.

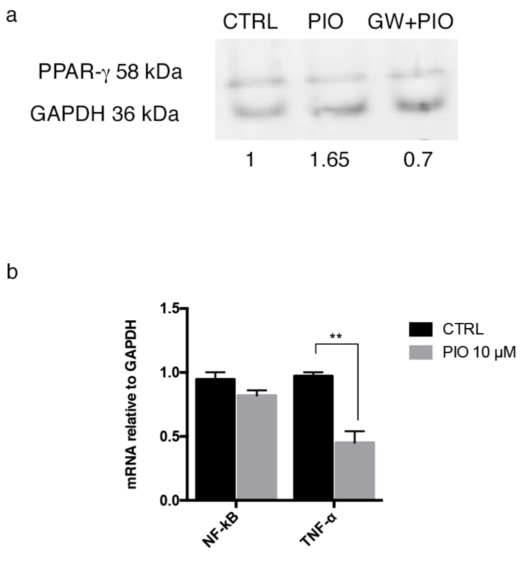

Figure S1. PPAR-γ expression in HUVEC. (a) Representative Western Blot for PPAR-γ detection in HUVEC treated with pioglitazone (10 μM) and pioglitazone (10 μM)/GW9662 (5 μM) combination for 24 h. Protein quantification was performed through densitometric analysis of band intensities by ImageJ and PPAR-γ/GAPDH ratio was reported. (b) Transcript levels of PPAR-γ target genes NF-kB and TNF-α, upstream regulators of the inflammatory process, by Real Time PCR. Statistical analysis was performed by paired Student's t test relative to untreated controls. **, $p < 0.01$. Abbreviations: HUVEC, human umbilical vein endothelial cells; CTRL, control; GAPDH, glyceraldehyde 3-phospate dehydrogenase; NF-kB, nuclear factor kappa-light-chain-enhancer of activated B cells; TNF-α, tumor necrosis factor α; PIO, pioglitazone; PPAR-γ, peroxisome-proliferator-associated-receptor-γ.

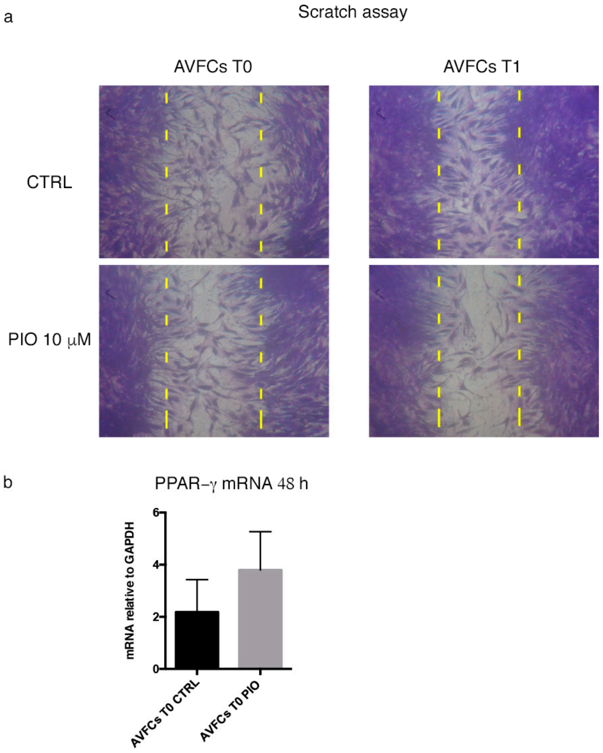

Figure S2: Effects of pioglitazone in AVFCs migration and PPAR-γ expression. (a) Representative images at 10 x magnification of cell migration performed by manual scratch assay in AVFCs T0 and AVFCs T1 exposed to pioglitazone (10 μM) for 48 h, and stained with crystal violet. (b) PPAR-γ mRNA expression evaluated by Real Time PCR in AVFCs T0 exposed to pioglitazone (10 μM) for 48 h. Results are expressed as fold changes relative to untreated controls and reported as mean ± SEM of at least three independent experiments (n=3). Abbreviations: AVFCs, arteriovenous fistula cells; CTRL, control; GAPDH, glyceraldehyde 3-phospate dehydrogenase; n, number of values; PIO, pioglitazone.
